# miR‐708/LSD1 axis regulates the proliferation and invasion of breast cancer cells

**DOI:** 10.1002/cam4.623

**Published:** 2016-02-02

**Authors:** Lin Ma, Shan Ma, Guimei Zhao, Longqiu Yang, Peng Zhang, Qingting Yi, Shuguang Cheng

**Affiliations:** ^1^ Department of NeurologyShanghai Tongji Hospital, Tongji UniversitySchool of MedicineShanghai200065China; ^2^ Department of OncologyThe Center Hospital of Zaozhuang Mining GroupZaozhuang277000China; ^3^ Vocational College of ZaozhuangZaozhuang277000China; ^4^ Department of AnesthesiologyZhengzhou Central Hospital Affiliated to Zhengzhou University195 Tongbai RoadZhengzhou CityHenan Province450007China

**Keywords:** Breast cancer, invasion, LSD1, miR‐708, proliferation

## Abstract

Breast cancer is one of the most common malignant tumors in women worldwide. The microRNAs (miRNAs) are small, noncoding RNAs that regulate various biological processes, including breast cancer. miR‐708 played an important role in a variety of cancers. However, its involvement in breast cancer remains largely unclear. In this study, we found that forced the expression of miR‐708 in breast cancer cell lines decreased cell proliferation and invasion, whereas inhibition of miR‐708 increased cell growth and invasion. miR‐708 could directly target the LSD1 3′UTR to downregulate the expression. Further studies suggested that inhibition of LSD1 could phenocopied function of the miR‐708 overexpression in MDA‐MB‐231 cells .Overexpression of LSD1 could counteract the effects of miR‐708 on the proliferation and invasion. Taken together, the results indicate that miR‐708 may function as a tumor suppressor gene in breast cancer development, and miR‐708/LSD1 axis may be a therapeutic intervention in breast cancer in the future.

## Introduction

Cancer is a serious threat to human health and social development. According to the report of the International Agency for Research on Cancer (IARC), there were about 1.67 million new cases of breast cancer in the world accounting for 11.88% of all cancer making it become the second most common malignancy worldwide. Breast cancer was regarded as the most serious malignancy that influenced physical and mental health for female 1. However, the regulatory mechanism of the initiation and the development remain largely unknown.

MicroRNAs (miRNAs), class of endogenous and noncoding RNA molecule, are about 21 nucleotides in length and commonly exist in plants, animals and virus genomes. It can directly bind to the 3′‐untranslated region (3′‐UTR) of target transcripts and degrade target mRNA level or suppress its translation in many kinds of biological processes 2, 3, 4, especially in cancer cell proliferation, migration, differentiation, and many other biological functions 5. The discovery of miRNAs has created a new area of cancer research and uncovered the complicacy of cancer biology 6. Iorio et al. firstly reported in 2005 that 29 miRNAs were significantly altered in breast tumor tissues compared to normal breast tissues. Recently, more and more miRNAs were found to be involved in the progression and development of breast carcinoma 7. MicroRNA‐708 (miR‐708), a newly discovered miRNA, has proved to be critical in the regulation of different kinds of tumor. Aberrant miR‐708 expression is closely associated with cell proliferation, migration, invasion, apoptosis, and patient prognosis in renal cell carcinoma, lung adenocarcinoma, hepatocellular carcinoma, ovarian carcinoma, bladder carcinoma, and prostate carcinoma 8, 9, 10, 11, 12, 13. However, few studies have paid attention to the functions of miR‐708 in breast carcinoma and the molecular regulation mechanism of miR‐708 in breast carcinoma still remains largely unknown.

Lysine‐specific histone demethylase 1 (LSD1) is a kind of histone demethylase which specifically demethylated mono‐ and dimethylated lysine 4 and lysine 9 of histone H3 14. LSD1 is necessary for mammalian development and is involved in various biological processes. Recent studies have demonstrated that LSD1, located in the nucleus, regulated gene transcription 15. LSD1 has been closely linked to embryonic development and tumorigenesis 16. Moreover, many evidences identified its pivotal role in breast carcinoma and suggested a possible molecular mechanism to some extent 17, 18, 19, 20, 21, 22, 23, 24, 25, 26. Nevertheless, as a novel and important epigenetic regulator, the further mechanism of the regulation of LSD1 and the upregulator of LSD1 still remain largely unknown.

In this study, we reported that forced expression of miR‐708 significantly suppressed MDA‐MB‐231 proliferation and invasion, whereas inhibition of miR‐708 promoted MDA‐MB‐231 proliferation and invasion. Furthermore, we identified LSD1 as a potential target gene of miR‐708. siRNA‐mediated knockdown of LSD1 could partially mimic the effects of miR‐708. Overexpression of LSD1 could abrogate miR‐708‐inhibited cell proliferation and invasion.

## Material and Methods

### Cell culture

The human breast carcinoma cell line MDA‐MB‐231 was purchased from Cell Bank at Shanghai Institutes for Biological Sciences of Chinese Academy of Sciences. Cells were cultured in Dulbecco's modified Eagle's medium (DMEM, Thermo, Waltham, MA, USA) supplemented with 10% fetal bovine serum (FBS, Gibco, Grand Island, NY, USA), 100 units/mL streptomycin, and 100 units/mL penicillin.

### Plasmid and transfection

The 3′UTR segment of wild‐type LSD1 mRNA, which possessed the binding site for miR‐708, was amplified and cloned into the pGL3 vector. The mutant miRNA‐binding sites were obtained by replacing the miRNA‐binding site sequence with miRNA seed sequences using the QuickChange Lightning Multi Site‐Directed Mutagenesis Kit (Agilent Technologies, Santa Clara, CA, USA). The LSD1 expression vector was performed by cloning LSD1‐coding sequence into Fugw. The miR‐708 precursor, precursor control, inhibitor, and inhibitor control were purchased from Ribobio (Guangzhou, China). Cells were harvested or suspended for RT‐PCR, western blotting, and other experiments mentioned in this paper.

### RT‐PCR for miRNA and mRNA expression

To measure the expression of miRNAs, 2 *μ*g total RNA was used to synthesize cDNA. Expression values were normalized to the control endogenous small RNA U6. To detect mRNA expression, 500 ng of total RNA was used to synthesize cDNA. Primer sequences for LSD1 was listed as follows: GAPDH (forward: 5′‐CTGGGCTACACTGAGCACC‐3′; reverse: 5′‐ AAGTGGTCGTTGAGGGCAATG‐3′), LSD1 (forward: 5′‐GTGGACGAGTTGCCACATTTC ‐3′; reverse: 5′‐TGACCACAGCCATAGGATTCC‐3′).

### Western blotting

Proteins were separated by SDS‐PAGE and transferred to membranes. Primary antibodies used in this study were listed as follows: anti‐GAPDH (ab97626, Abcam, Cambridge, MA, USA), anti‐LSD1 (ab17721, Abcam, Cambridge, MA, USA). Signals were detected by using enhanced chemiluminescence (ECL) (Thermo, USA).

### Cell proliferation assay

Cells were transfected with precursor or inhibitor for 48 h and then seeded in 96‐well plates at a density of 3.5 × 10^3^ cells/well. Cell counts were conducted after 24 h, 48 h, and 72 h. Cell counting Kit‐8 solution was added at each time point for 1 h at 37°C and measured the absorbance at 490 nm with a spectrophotometer.

### Cell invasion assay

Cell invasion assay was performed by using transwell inserts with 8.0 *μ*m pores in 24‐well plates. Transwell filter was coated with Matrigel to mimic the microenvironment. Cells were suspended in medium without serum. In the bottom chamber, the culture medium was added with serum as a chemoattractant. Cells (4 × 10^4^) were seeded on the upper side of the insert. Then the chambers were incubated for 20 h and removed the cells that did not migrate through the filter with cotton swabs. Four percent of paraformaldehyde was used to fix the cells on the lower side of the insert. Finally, Hoechst 33342 was used to stain cells. We chose four different fields under microscopic vision to count the cells and calculate the mean.

### Luciferase reporter assay

293FT cells were used to study the relationship between miR‐708 and LSD1. For each 24‐well plate, 0.3 nmol/L pre‐miR‐708 or control pre‐miR was transfected. 200 ng of the firefly luciferase report vector and 5 ng of the control vector containing renilla luciferase were transfected in the meantime. Forty‐eight hours after transfection, the luciferase activities were detected using the Dual‐Luciferase Reporter Assay System (Promega, Fitchburg, WI, USA).

### Statistical analysis

All statistical analyses were carried out using SPSS 18.0 (IBM, Armonk, NY, USA). Statistical significance was determined using a Student's *t*‐test. Values were presented as the mean ± SD.*,**,*** means *P* < 0.05, *P* < 0.01, *P* < 0.001, respectively.

## Results

### miR‐708 inhibited tumor cell proliferation and invasion

**Figure 1:**
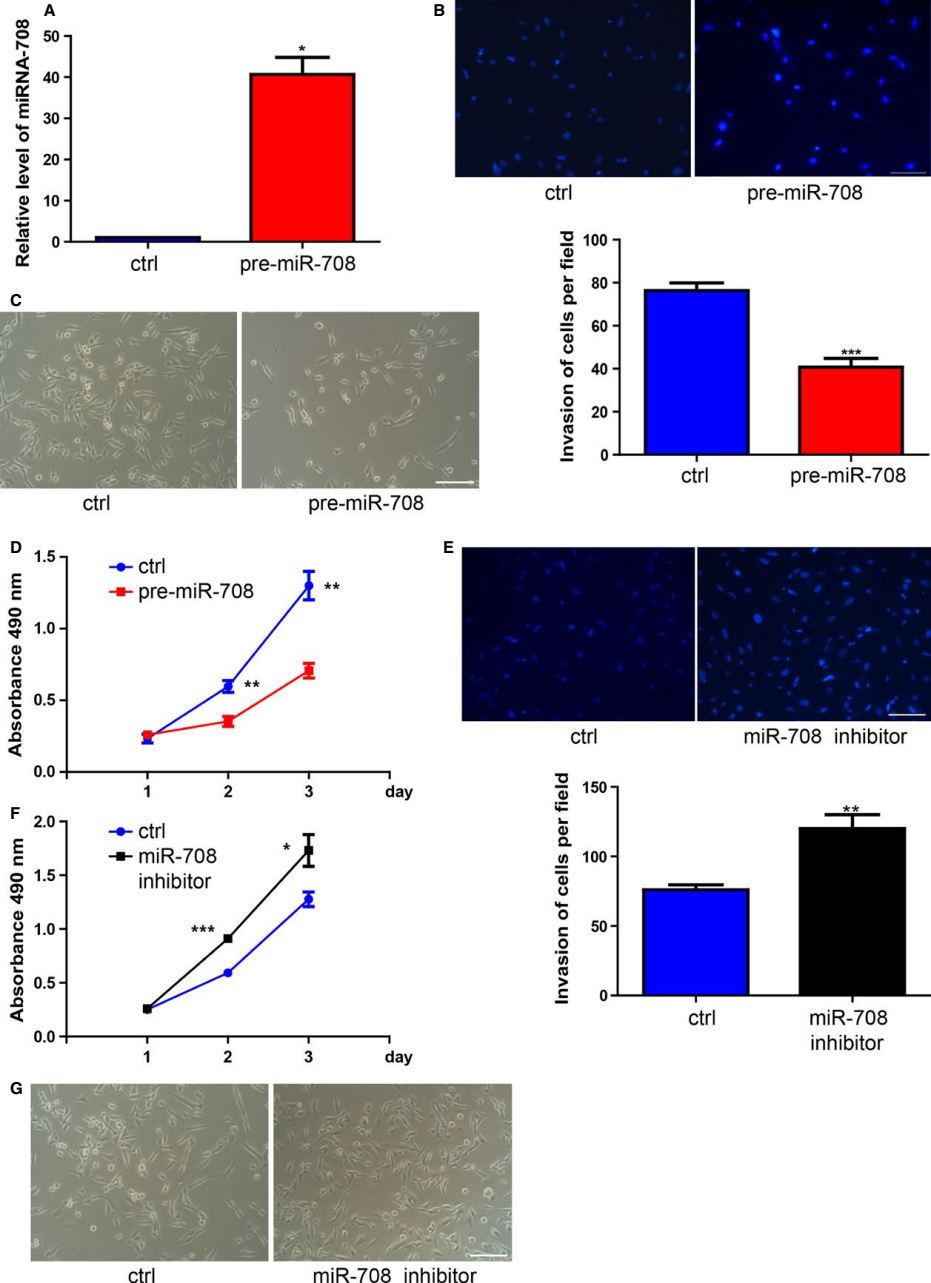
miR‐708 has a positive correlation with breast cancer cells growth and invasion. (A). Transfection efficiency was confirmed at 48 h by using RT‐PCR. *P < 0.05.(B). Overexpression of miR‐708 apparently inhibited MDA‐MB‐231 cells invasion, whereas inhibition of miR‐708 obviously promoted its invasion. **P < 0.01, ***P < 0.001. (C). Morphology of MDA‐MB‐231 cells at 48 h after plating the same number cells in the dish (left: transfected with pre‐miR‐control, right: transfected with pre‐miR‐708). (D). CCK‐8 assay was performed to measure the growth rate of MDA‐MB‐231 cells upon pre‐miR‐708. (E). Inhibition of miR‐708 promoted MDA‐MB‐231 cells invasion **P < 0.01. (F). Inhibition of miR‐708 promoted MDA‐MB‐231 cells proliferation. *P < 0.05. (G). Morphology of MDA‐MB‐231 cells at 48 h after plating the same number cells in the dish (left: transfected with Inhibitor control, right: transfected with miR‐708‐inhibitor). *P < 0.05.

To determine the role of miR‐708 in breast cancer cells, we use MDA‐MB‐231, a highly malignant degree cell line and a useful tool for studying tumorigenesis mechanism and biology 27, 28. Transient transfection of miR‐708 precursor resulted in miR‐708 overexpression as validated by RT‐PCR (Fig. 1A). Interestingly, we observed apparent decrease in growth rate when miR‐708 was upregulated (Fig. 1C). miR‐708‐inhibitior cell grew significantly slower than the control cell line (Fig. 1G). Next, we quantified cell growth rate by using CCK8 assay and found that miR‐708 had a negative correlation with cell proliferation (Fig. 1D, F). Moreover, our results found that miR‐708 was involved in cell invasion of breast cancer. Overexpression of miR‐708 decreased MDA‐MB‐231 invasion, whereas inhibition of miR‐708 promoted MDA‐MB‐231 invasion (Fig. 1B, E).

### miR‐708 specifically regulated LSD1 expression in breast cancer

**Figure 2:**
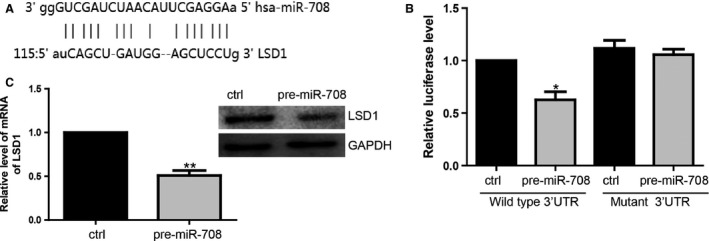
miR‐708 specifically targets LSD1. (A). MiR‐708 target site in the LSD1 3′UTR. (B). Luciferase activity of WT plasmid was suppressed in the present of pre‐miR‐708. *P < 0.05. (C). Overexpression of miR‐708 downregulated LSD1 expression at mRNA and protein level. **P < 0.01.

To identify a target gene of miR‐708, TargetgScan, Pictar, and miRanda were used to search for mRNAs that interact with miR‐708. We focused on LSD1, inspired not only by the program prediction, but also by the importance of LSD1 in breast cancer. The predicted binding site between LSD1 and miR‐708 was showed in Figure 2A. Then, we performed luciferase reporter assay to determine whether miR‐708 can target the 3′UTR of LSD1. Wide type and mutant 3′UTR reporter was generated and transfected into 293FT cells with pre‐miR‐708. Our results suggested that the luciferase activity of WT‐3′UTR was obviously suppressed by pre‐miR‐708 (Fig. 2B). Then we found endogenous LSD1 was regulated by pre‐miR‐708. Both mRNA and protein level of LSD1 were decreased after miR‐708 transfection (Fig. 2C).

### LSD1 was critically involved in the regulation of proliferation and invasion in breast cancer

**Figure 3:**
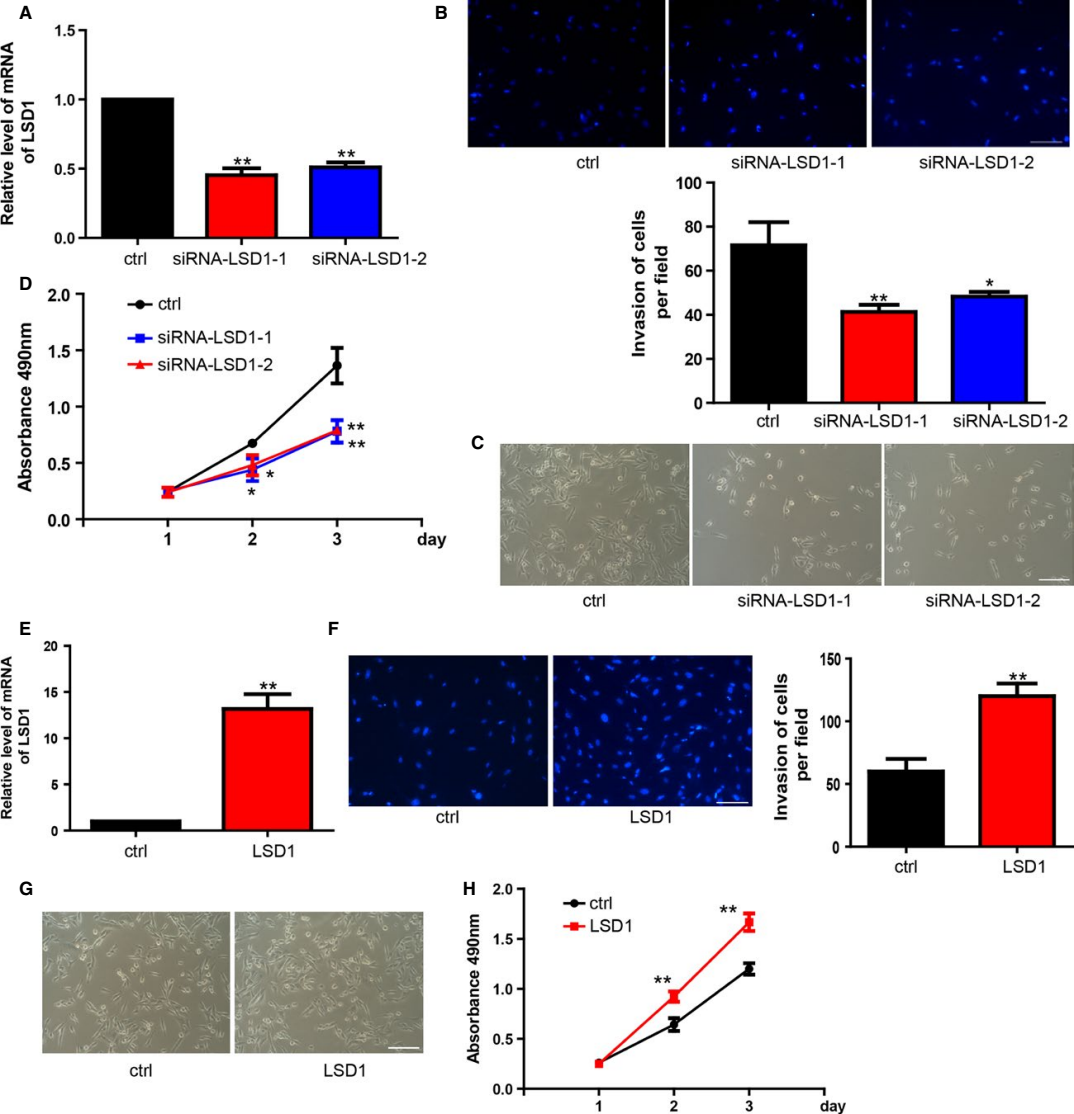
LSD1 inhibition partially phenocopies miR‐708 overexpression, whereas overexpression of LSD1 has opposite effects. (A). The mRNA level of LSD1 was reduced by siRNA‐LSD1‐1 or siRNA‐LSD1‐2 which specifically target LSD1. **P < 0.01. (B). Inhibition of LSD1 upregulated MDA‐MB‐231 cells invasion. *P < 0.05, **P < 0.01. (C). MDA‐MB‐231 cells grew much faster as a result of LSD1 inhibition. (D). Proliferation of MDA‐MB‐231 cells was analyzed using CCK‐8 assay. **P < 0.01. (E). LSD1 expression was upregulated with the treatment of LSD1 lentivirus infection. **P < 0.01. (F). Lentivirus infection efficiency was confirmed at 72 h by using RT‐PCR. **P < 0.01. G. Cells grew much slower with the treatment of LSD1 lentivirus infection. H. CCK‐8 assay was performed to evaluate the proliferation rate of MDA‐MB‐231 cells with the treatment of LSD1 lentivirus infection. **P < 0.01. *P < 0.05.

In view of the observed functions of miR‐708 on proliferation and invasion, we tried to find whether miR‐708 regulated cancer cell through LSD1. Two different LSD1 siRNA were transfected into cancer cell. RT‐PCR was conducted to confirm the silence effects of siRNA (Fig. 3A). The data revealed that, as expected, LSD1 siRNA‐transfected cells inhibited cell invasion, which is consistent with the effects of miR‐708 (Fig. 3B). Moreover, treatment with LSD1 siRNA also resulted in decreased proliferation, which is similar to the phenomenon observed upon miR‐708 overexpression in cancer cells (Fig. 3C–D). LSD1 lentivirus vector was constructed and expression efficiency was measured by RT‐PCR (Fig. 3E). Overexpression of LSD1 could significantly enhance MDA‐MB‐231 proliferation and invasion (Fig. 3F–H).

### miR‐708 inhibited proliferation and invasion by inhibiting LSD1

**Figure 4:**
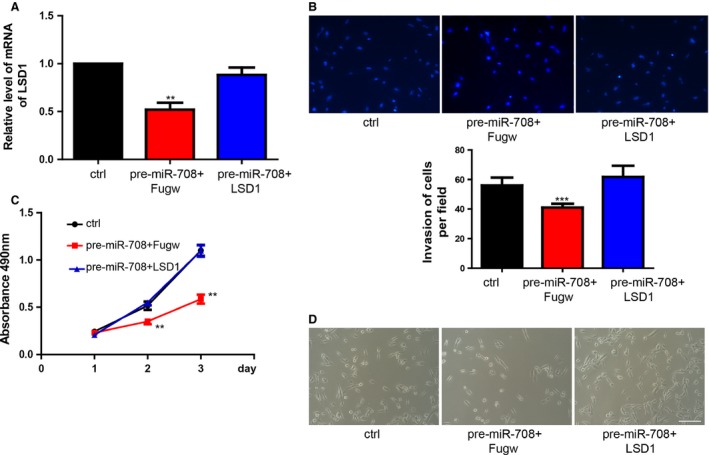
Overexpression of LSD1 could rescue miR‐708‐inhibited proliferation and invasion. (A). The expression of LSD1 with the treatment of LSD1‐Fugw lentivirus or control‐Fugw. Data shown are means ± SD (n=3), **P<0.01. (B). Overexpression of LSD1 could rescue miR‐708‐inhibited cell invasion. Data shown are means ± SD (n=3), ***P<0.001. (C). Proliferation rate of MDA‐MB‐231 cells treated with LSD1‐Fugw lentivirus or control lentivirus was measured by MTS assay. (D). Cells treated with pre‐miR‐708 and Fugw lentivirus grew slower compared with control group, however, LSD1 overexpression could rescue the inhibitory function of pre‐miR‐708.

To determine whether the miR‐708‐LSD1 interaction is necessary for breast cancer cell, MDA‐MB‐231 was infected with lentivirus vector that overexpressed LSD1. The cells were subsequently transfected with pre‐miR‐708. LSD1 expression in different groups was showed in Figure 4A. We found that overexpression of LSD1 could partially counteract miR‐708‐decreased invasion (Fig. 4B). Moreover, overexpression of LSD1 could partially abrogated miR‐708‐induced proliferation (Fig. 4C–D).

## Discussion

Preventing tumor growth and invasion has been the most challenging problem in the treatments for breast cancer. Our data showed that overexpression of miR‐708 significantly decreased MDA‐MB‐231 invasion, whereas inhibition of miR‐708 increased MDA‐MB‐231 invasion. Previous studies have revealed the important role of miR‐708 in regulating cancer cell migration and invasion in other malignancies. miR‐708 expression was decreased in human renal cell carcinoma (RCC) specimens and downregulation of miR‐708 in RCC cell lines increased cancer cell invasion, which is similar to our finding 8. Besides, overexpression of miR‐708 inhibited cell invasion in the human glioblastoma cell lines A172 and T98G 29. However, downregulation of miR‐708 in hepatocellular cell suppressed tumor invasion and migration 13. miR‐708 overexpression in lung cancer cell lines increased cell invasion by twofold compared with the control 9. This suggests that miR‐708 have divergent functions in different microenvironments. Additionally, we found that miR‐708 had a negative relation with cell proliferation in breast cancer. Inhibition of miR‐708 in MDA‐MB‐231 could enhance cell proliferation, whereas restoration of miR‐708 with mimics could reduce cell proliferation. Several studies have reported on the dysregulation of miR‐708 in cell proliferation 9, 29, 30. Collectively, our results demonstrated that miR‐708 function as an antitumor factor in breast cancer.

Up to now, an increasing number of miRNAs have been reported to be closely related to breast cancer. miR‐10b promoted breast cancer cell migration and invasion by inhibiting HOXD10 protein synthesis 31. miR‐21 overexpression in breast cancer is causally linked to advanced clinical stage, lymph node metastasis, and patient poor prognosis. Moreover, miR‐21 silencing in breast cancer cells suppressed cell growth and tumor suppressor protein Programmed Cell Death 4 (PDCD4) is a functionally important target for miR‐21 in breast cancer 32, 33. MiR‐155 served as an oncomiR in breast cancer in promoting the proliferation of breast cancer cells 34. Besides miRNAs mentioned above, miR‐451, miR‐146, miR‐221/222, and other miRNAs were also confirmed to be closely associated with breast cancer 35, 36, 37.

In breast cancer cell line MDA‐MB‐231, using western blotting and luciferase assay, we found miR‐708 directly binds to the 3′utr region of LSD1, a critical gene that has been widely verified to be involved in breast cancer metastasis 19, 22, 23, 38. Our data showed that siRNA‐mediated silencing of LSD1 could partially mimic the effects of miR‐708, whereas restoration of LSD1 could counteract the effects of miR‐708 in MDA‐MB‐231. The results demonstrated that miR‐708 and LSD1 closely interact in regulating growth and invasion of breast cancer cells.

A few of studies are currently ongoing to make a foundation for introducing miRNAs in clinical practice. A phase I trial is underway in United Stated to test MRX34 (Mirna Therapeutics, Inc., Austin, TX, USA) as the first miRNA replacement therapy in human. Given that the role of miR‐708/LSD1 as genomic regulators in breast cancer development and metastasis, miR‐708 might be applied to treatment for breast cancer,

In conclusion, our findings showed that miR‐708 might serve as an antioncogene by directly targeting LSD1, through promoting cell growth and cell invasion. Hence, it suggests the potential application of miR‐708/LSD1 axis for treatment of breast cancer in the future.

## Conflict of Interest

None declared.

## Supporting information


**Figure S1.** Related to the Figure 1 miR‐708 has a positive correlation with breast cancer cells growth and invasion.

**Figure S2.** Related to the Figure 3 LSD1 regulates the proliferation and invasion of T47D cells.

**Figure S3.** Related to the Figure 4 LSD1 directly blocked the function of miR‐708 on inhibiting proliferation and invasion of T47D cells.
