# Supplementary figures and images for: miR‐708/LSD1 axis regulates the proliferation and invasion of breast cancer cells
Source: Cancer Med. 2016 Feb 2;5(4):684–92. doi: 10.1002/cam4.623 (PMC4831287; doi:10.1002/cam4.623)

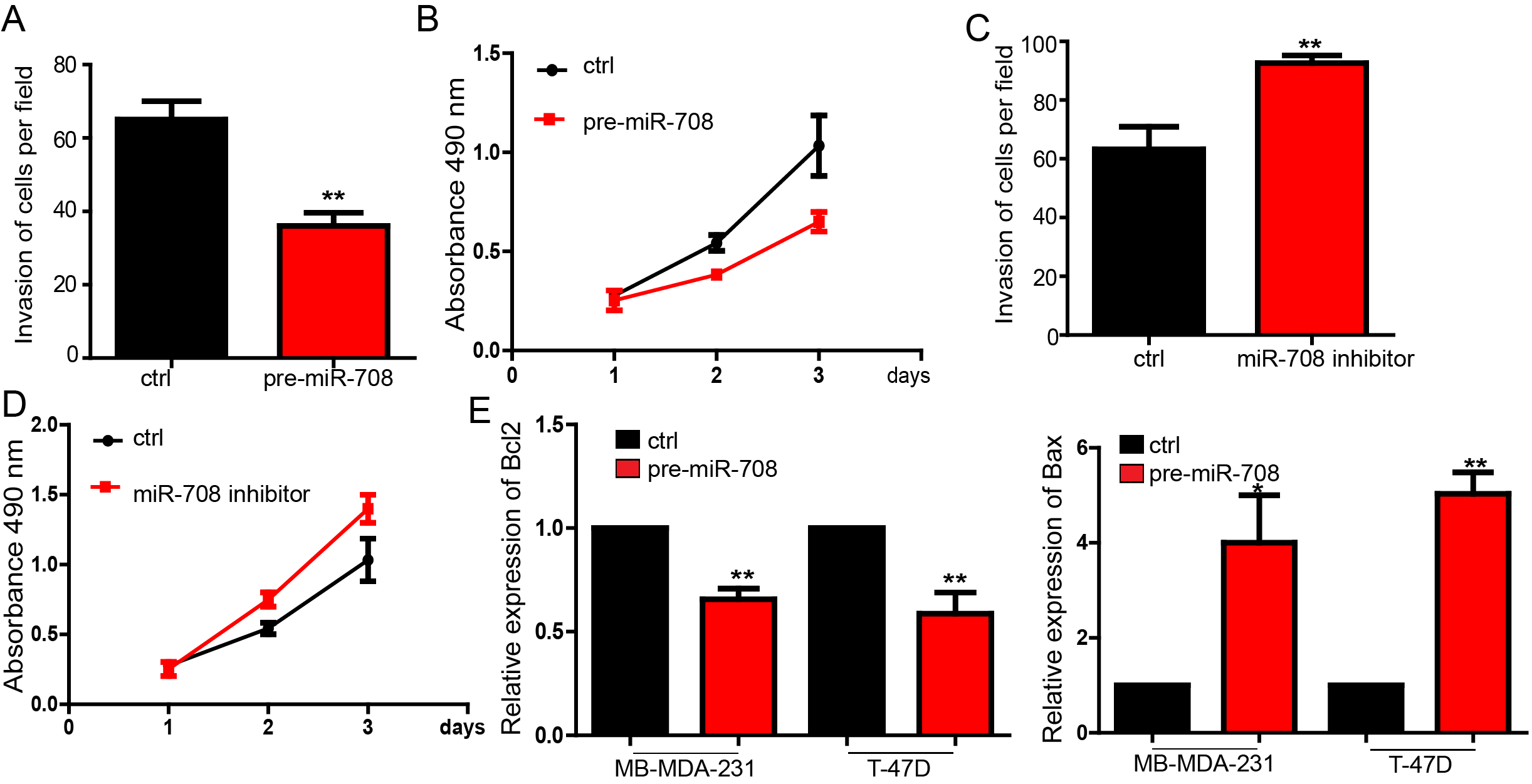

Supplement: Supplementary file 1 — Figure S1. Related to the Figure 1 miR‐708 has a positive correlation with breast cancer cells growth and invasion. [file CAM4-5-684-s001.tif]

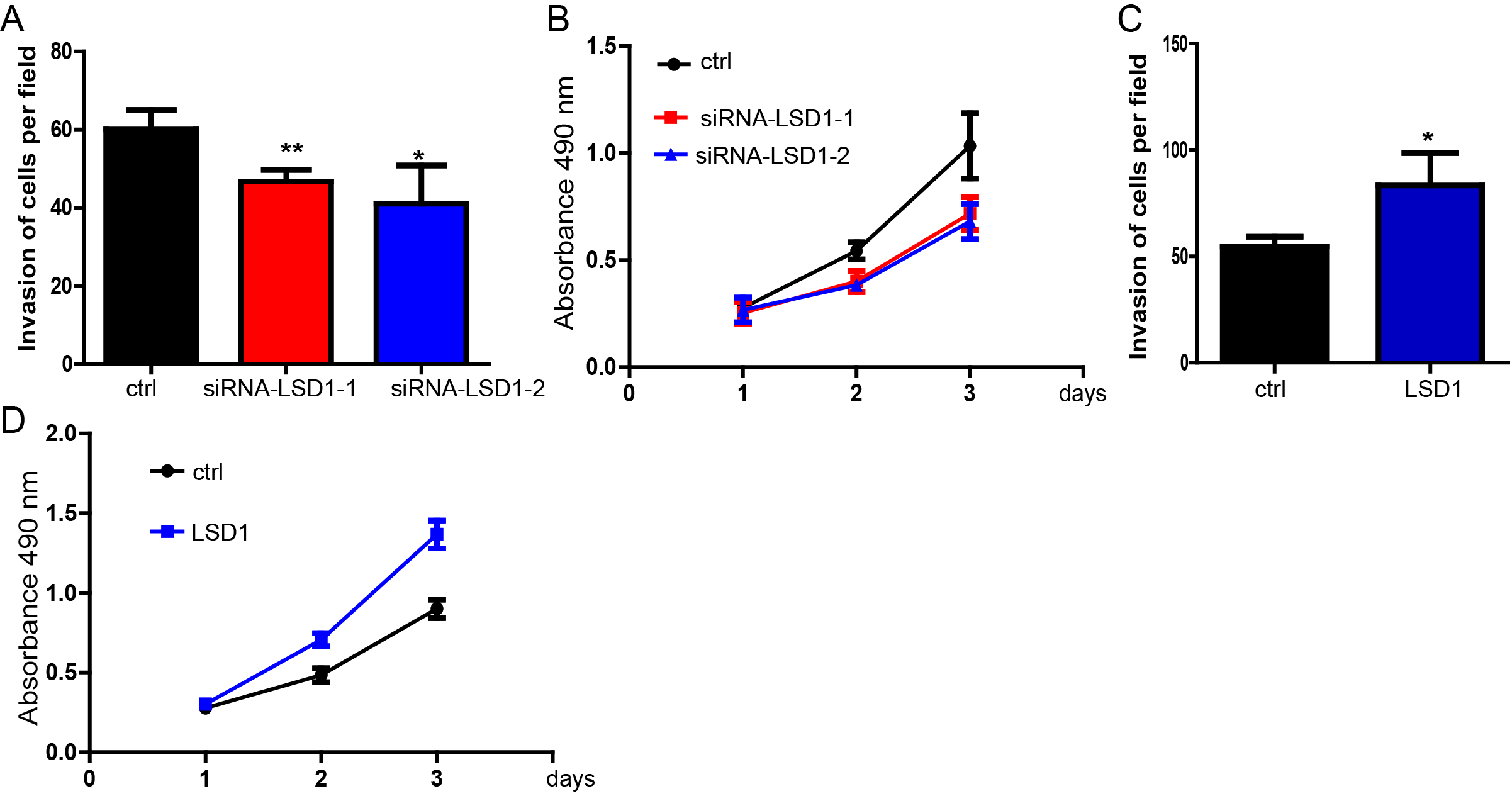

Supplement: Supplementary file 2 — Figure S2. Related to the Figure 3 LSD1 regulates the proliferation and invasion of T47D cells. [file CAM4-5-684-s002.tif]

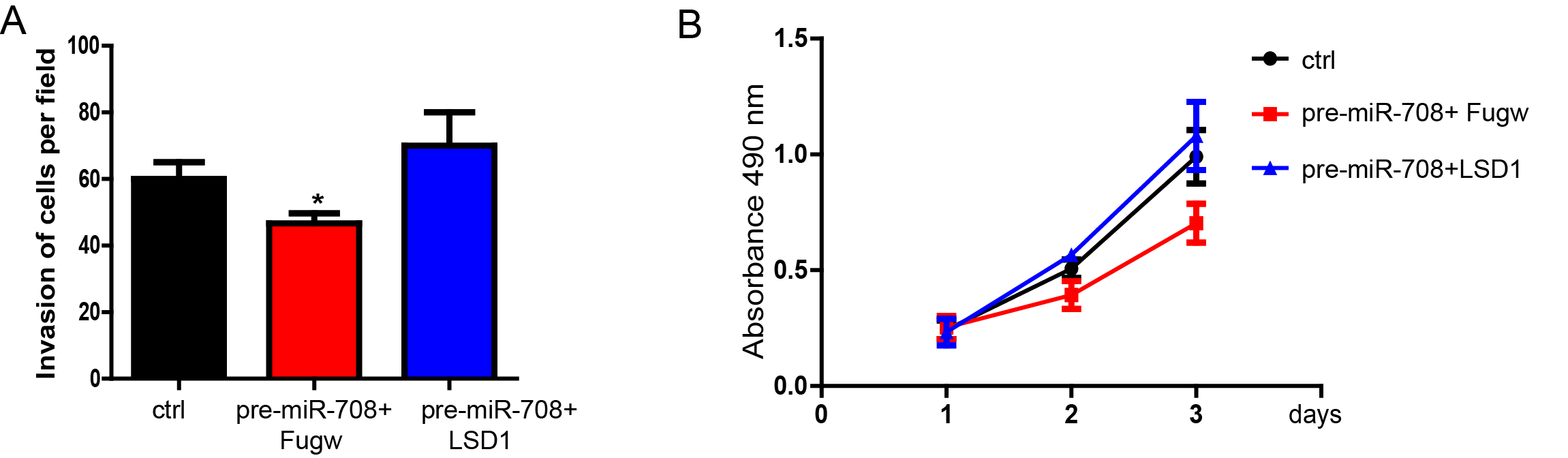

Supplement: Supplementary file 3 — Figure S3. Related to the Figure 4 LSD1 directly blocked the function of miR‐708 on inhibiting proliferation and invasion of T47D cells. [file CAM4-5-684-s003.tif]
